# Nestedness in Arbuscular Mycorrhizal Fungal Communities in a Volcanic Ecosystem: Selection of Disturbance-tolerant Fungi along an Elevation Gradient

**DOI:** 10.1264/jsme2.ME19073

**Published:** 2019-08-14

**Authors:** Rifa Atunnisa, Tatsuhiro Ezawa

**Affiliations:** 1 Graduate School of Agriculture, Hokkaido University Sapporo, Hokkaido 060–8589 Japan

**Keywords:** community structure, volcanic slope, nestedness, soil disturbance, selection pressure

## Abstract

Arbuscular mycorrhizal (AM) fungi play a significant role in the establishment and resilience of vegetation in harsh environments, such as volcanic slopes, in which soil is frequently disturbed by ash falling and erosion. We characterized AM fungal communities associated with a pioneer grass in a volcanic slope based on the disturbance tolerance of the fungi, addressing the hypothesis that soil disturbance is a major ecological filter for AM fungi in volcanic ecosystems and, thus, fungi that are more tolerant to soil disturbance are selected at higher elevations (*i.e*. nearer to the crater). Paired soil-core samples were collected from the rhizosphere of *Miscanthus sinensis* between the vegetation limit and forest limit on a volcanic slope and used in a trap culture with *M. sinensis* seedlings, in which one of the paired samples was sieved to destroy hyphal networks (disturbance treatment), while the other was not (intact treatment). Seedlings were grown in a greenhouse for two months, and the roots were subjected to molecular analysis of fungal communities. AM fungal diversity decreased with increasing elevations, in which nested structure was observed. Community dissimilarity between the disturbed and intact communities decreased with increasing elevations, suggesting that communities at higher elevations were more robust against soil disturbance. These results suggest that AM fungi that are more tolerant to soil disturbance are more widely distributed across the ecosystem, that is, they are generalists. The wide distribution of disturbance-tolerant fungi may have significant implications for the rapid resilience of vegetation after disturbance in the ecosystem.

The slopes of active volcanoes are a harsh habitat for plants; soil disturbance due to ash falling and erosion are frequent, and, thus, vegetation is generally poor, particularly near the crater (*i.e*. at higher elevations). The protection of volcanic slopes by vegetation is important for the stabilization of soil, particularly after a large eruption, which may reduce the risk of catastrophic debris flow. However, members of primary vegetation on volcanic slopes are limited within those that have disturbance-tolerant traits due to the frequent disturbance of the habitat. *Miscanthus sinensis* Anders. is a pioneer grass species that is a common member of primary vegetation in eastern Eurasia and South-East Asia and also common in volcanic slopes (3, 21, 26).

Pioneer plants have developed various strategies to adapt to harsh environments, and one distinct strategy is the association with arbuscular mycorrhizal (AM) fungi. AM fungi associate with 70–80% of land plants in all major biomes (8) and enhance the growth of host plants by enhancing the uptake of mineral nutrients, particularly phosphorus (41). Many pioneer species in early primary succession on volcanic slopes (14, 43) and at high elevations (11, 28, 30, 31, 42) form AM associations. It has been demonstrated that the fungi play a significant role in the establishment of pioneer vegetation; *e.g*., *M. sinensis* and the legume pioneer *Lespedeza cyrtobotrya* are highly dependent on AM associations for their establishment in acidic soil (33).

The mechanical destruction of hyphal networks by soil disturbance has a significant impact on AM fungal communities (20, 22, 29, 39), implying that disturbance functions as an ecological filter for selecting the fungi that are tolerant to hyphal destruction. In volcanic ecosystems, severity of disturbance may increase with increasing elevation towards the crater (10). Therefore, AM fungi that are more tolerant to disturbance may preferentially inhabit higher elevations, whereas those that are sensitive to disturbance may be more abundant at lower elevations at which disturbance are less severe.

In the present study, we hypothesized that soil disturbance is a major ecological filter for AM fungal communities in volcanic ecosystems, and, thus, the fungi that are tolerant to soil disturbance are selected at higher elevations (*i.e*. nearer to the crater). To test this hypothesis, we employed the soil trap culture approach and applied artificial disturbance to soil samples collected from the rhizosphere of the pioneer grass *M. sinensis* along an elevation gradient in a volcanic slope.

## Materials and Methods

### Sampling site

**Fig. 1:**
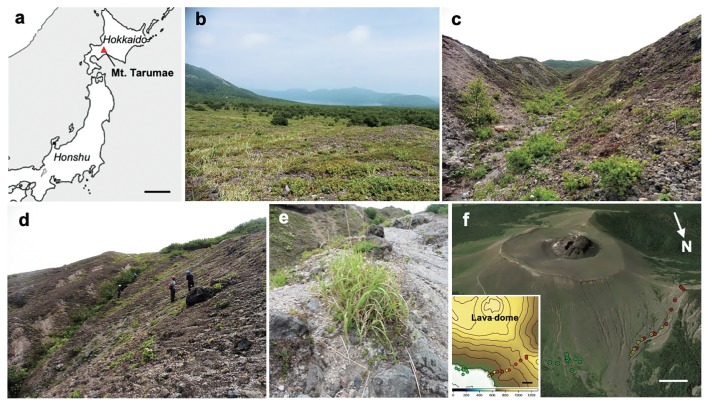
a, Location of Mt. Tarumae in Japan. Scale, 200 km. Three habitat types in the mountain: b, grassland; c, gully; d, slope. e, *Miscanthus sinensis* grown on a slope. f, Sampling points; green circles, grassland; yellow circles, gully; red circles, slope. Scales, 200 m. 3D- and 2D-maps were generated by Google Earth (https://www.google.co.jp/intl/ja/earth/) and GMT 5.4.5 (http://gmt.soest.hawaii.edu/), respectively, using the GPS data of sampling points (Table S1). The colors in the 2D map represent elevations.

Mt. Tarumae is located in the southeastern rim of Shikotsu caldera (42°41′26″N/141°22′36″E) in Shikotsu-Toya National Park, Hokkaido, Japan (Fig. 1a). The volcano occurred approximately 9,000 years ago, and the largest explosive eruptions that affected the Northern Hemisphere temperature were recorded in 1667 and 1739 (7). The latest eruption with ash falls and mudflows occurred between 1978 and 1981. In 2001 and 2003, the temperature of the crater reached more than 600°C, an sand and mud were emitted from the crater (15). Its height is 1,041 m a.s.l., and there is a lava dome (120 m in height) in the crater. The upper limit of the forest is approximately 630 m a.s.l., and above the limit, *M. sinensis* is distributed into three types of habitats, grassland, gullies, and slopes, on the north-facing slope. The grassland is an open flat area (inclination angles are less than 10°) located at 630–660 m a.s.l., at which *M. sinensis* and the shrub *Ledum palustre* subsp. *diversipilosum* var. *diversipilosum* largely dominate (Fig. 1b). In addition to the two dominant species, large colonies of *Spiraea betulifolia* var. *betulifolia* and *Weigela middendorffiana* are patchily distributed. *M. sinensis* grown in this habitat generally develop a large clump of multiple stems (50–150 cm in diam). Above the grassland (up to the vegetation limit at 850 m a.s.l), several gullies (0.3–2 m in depth, inclination angles up to 15°) have developed along the slope of which the inclination angles are 20–40°. Water availability in the gullies appears to be higher than in the grassland and on the slope, particularly during spring when snow melts. Although the gullies and slopes are not fully covered with vegetation, plant diversity is markedly higher than in the grassland; *Alnus maximowiczii*, *Sorbus sambucifolia*, *Fallopia sachalinensis*, *Pennellianthus frutescens*, and *Salix reinii* are patchily distributed in addition to *M. sinensis*, *L. palustre* subsp. *diversipilosum* var. *diversipilosum*, and *S. betulifolia* var. *betulifolia* (Fig. 1c and d). *M. sinensis* that grows in the gullies and on the slopes generally forms smaller clumps than those in the grassland (Fig. 1e).

### Soil sampling

Ten individuals of *M. sinensis* were selected from each of the three habitats, grassland, gullies, and slopes (30 plants in total), along the north-facing slope towards the crater (Fig. 1f). After measuring the colony diameter of *M. sinensis*, two rhizosphere soil samples (*i.e*. paired-soil samples), including root fragments, were collected from each individual with a stainless steel core sampler (5×5 cm, 100 mL in vol) in June 2015 (14 samples) and August 2016 (16 samples) as AM fungal inocula for the soil trap culture (Table S1). In addition to these paired-soil samples, approximately 100 g of root-zone soil was collected from each individual for chemical analysis. Twenty kilograms of bulk soil was also collected where no vegetation was present (800 m a.s.l.) as a base medium for the trap culture. The chemical properties of the soil samples were analyzed at Tokachi Federation of Agricultural Cooperatives, Obihiro, Japan (Table S1).

### Disturbance treatment in the soil trap culture

One of the paired soil-core samples was excised from the core, sieved on a 2-mm stainless steel mesh to destroy hyphal networks of the fungi, and designated as a disturbed inoculum, whereas the other sample was carefully taken from the core sampler and used without sieving as an intact inoculum. The intact or disturbed inoculum (*ca*. 100 mL) was transferred to 350-mL plastic pots that were filled with 200 mL of the base medium (*i.e*. autoclaved bulk soil). Seeds of *M. sinensis* (Kaisesha, Otofuke, Japan) were sown onto the soil surface, covered with a thin layer of autoclaved river sand, grown only with tap water in a temperature/light/humidity-controlled greenhouse (day/night temperature 26/20°C; 14-h day length; 60% relative humidity), and thinned to 10 plants per pot two weeks after sowing. Two months later, the roots were harvested, washed with tap water, cut into 1-cm segments, randomized in water, blotted on a paper towel, frozen at −80°C for more than 1 d, freeze-dried for 2 d, and stored at −30°C for DNA extraction.

### Molecular identification

Between 10 and 20 mg of the freeze-dried root sample was transferred to a 2.5-mL tube with an O-ring sealed cap (Yasui Kikai, Osaka, Japan), ground using Multi-Beads Shocker (Yasui Kikai) with a metal cone at 2,500 rpm for 2×60 s, and DNA was extracted with DNeasy Plant Mini Kit (Qiagen, Tokyo, Japan) according to the manufacturer’s instructions. The divergent domain 2 of large-subunit ribosomal RNA gene (LSU rDNA) was amplified in a 25-μL reaction mixture of Expand High-Fidelity PLUS PCR System (Roche Diagnostics, Tokyo, Japan), 0.5 nmol μL^−1^ each of the fungus-specific primers, FLd3 (forward) and FLR2 (reverse) to which TruSeq-type forward- and reverse-adapter sequences (Illumina, Tokyo, Japan), respectively, were linked at the 5′-end (35), and 1 μL template DNA using C1000 Touch™ Thermal Cycler (BIO-RAD, Tokyo, Japan) with the following program: initial denaturation at 94°C for 2 min, followed by 30 cycles of denaturation at 94°C for 15 s, annealing at 48°C for 40 s, and polymerization at 72°C for 1 min, and final elongation at 72°C for 10 min. PCR products were purified with Agencourt AMPure XP SPRI magnetic beads (Beckman Coulter, Tokyo, Japan) and sequenced on the Illumina MiSeq platform (2×300 bp) at Bioengineering Lab (Atsugi, Japan). Nucleotides with a quality value (QV) <20 at the 3′ end and the adapter-index sequence at the 5′ end were trimmed, and those shorter than 40 bp were excluded. After quality filtering, paired-end reads (read 1 and read 2) were merged with a minimum overlap length of 10 nt using FLASH (http://ccb.jhu.edu/software/FLASH/). The merged sequence reads were subjected to BLASTn searches against the fungal LSU rDNA database consisting of 82,494 operational taxonomic units (OTUs) of fungi, including 412 OTUs of AM fungi, and assigned to the OTUs at a criterion of ≥95% similarities over 330 bp with an E value ≤10^−100^, which was executed in the open web interface “Arbuscular mycorrhizal fungi classification pipeline” (http://amfungi.kazusa.or.jp) (35).

### Statistical analysis

Total sequence reads assigned to AM fungal OTUs were standardized to 10^4^ reads per sample by resampling for community analysis. All statistical analyses were performed on R 3.2.3 platform (38). Shannon-Wiener indices, Bray-Curtis dissimilarity indices, Mantel test (9,999 permutations), and permutational multivariate analysis of variance (PERMANOVA) (9,999 permutations) were performed with the vegan package (Oksanen, J., F.G. Blanchet, M. Friendly, *et al*. 2013. vegan: Community Ecology Package. R package version 2.4–5. https://CRAN.R-project.org/package=vegan). In Mantel test, Bray-Curtis dissimilarity index was employed as a measure of community distance.

Community nestedness was evaluated using nested overlap and decreasing fill (NODF) as an index (2), in which the hypothesis that species (OTU)-poor communities are subsets of species (OTU)-rich communities was tested. In this analysis, the OTU composition data of the intact and disturbed communities from the paired soil samples were combined and transformed to presence-absence data, and a maximally stacked matrix, in which the orders of the columns (soil samples) and rows (OTUs) were sorted by OTU richness and the occurrence of OTU (number of soil samples in which the OTU was detected), respectively, was then constructed. A two-sided test was performed using the function oecosimu in the vegan package, in which the significance of NODF among columns (NODF_column_) was tested against 1,000 randomly generated matrices (Monte Carlo procedure) under the column-total fixed null model that assumes the number of species in each sample remains constant. The index ranged between 0 (non-nested) and 100 (fully nested) and was deemed significant if the value was higher than the expected value obtained from randomly generated matrices. The Z-scores of NODF_column_ were calculated as (NODF_column_–NODF_column-exp_)/SD_exp_, where SD_exp_ is the standard deviation of the expected values. A significant positive Z-score indicates that there was a nested pattern in the matrix, while a significant negative Z-score indicates anti-nestedness.

Individual OTUs were characterized with respect to soil disturbance based on gain and loss of the sequence reads by the disturbance treatment relative to those in the intact treatment. All read number data were initially transformed to logarithmic values (log_10_) according to Anderson *et al.* (4), and the ‘responsiveness’ and ‘robustness’ of *OTU_j_* were calculated by the following equations:

- $Responsivenss\;of\;OTU_{j}=\frac{D_{j}-I_{j}}{D_{j}+I_{j}}$
- $Robustness\;of\;OTU_{j}=1-\left|\frac{D_{j}-I_{j}}{D_{j}+I_{j}}\right|$

where *D_j_* and *I_j_* are the total read numbers of *OTUj* detected in the disturbed and intact treatments, respectively, across all samples. The former index (a) of the OTUs that gained and lost sequence reads by disturbance shows positive and negative scores, respectively, whereas the latter index (b) of the OTUs that showed large and small differences in sequence-read abundance between the disturbance and intact treatments indicates low and high scores, respectively, between 0 and 1.

## Results

### Assignment to OTUs of arbuscular mycorrhizal fungi

PCR products were successfully obtained from all samples, and the sequencing of these products generated 70,000–170,000 paired-end reads that were merged into 48,000–130,000 sequences. Among them, 41 and 8% on average were assigned to the OTUs of AM fungi (*i.e*. glomeromycotinan fungi) (Table S2 and S3) and those of non-glomeromycotinan fungi (data not shown), respectively. The rest of the reads were not assigned to any of the OTUs in the database. Increases in AM fungal diversity by subsequent BLASTn searches of these unassigned sequences against the GenBank database were only marginal (data not shown) and were not reflected in the results. Rarefaction curves of AM fungal OTU richness were constructed based on both the numbers of samples and sequence reads to assess the sampling effort status (Fig. S1). Both curves showed leveling off, suggesting that our sampling provided reasonable coverage of AM fungal diversity at the site.

### Environmental factors that drive communities

Environmental factors employed for community analysis are listed in Table S4. Pairwise correlation analysis between these factors indicated that elevations did not correlate with any of the plant or soil factors, whereas the colony diameter of *M. sinensis* positively correlated with NH_3_-nitrogen. Exchangeable bases generally positively correlated with each other and negatively with pH. Among the three habitats, the colony diameter was larger in the grassland and gully habitats than in the slope habitat (Table S5). pH was the lowest in the slopes and highest in the gullies, while soil phosphate, exchangeable bases, and NH_3_-nitrogen were generally higher in the grassland and slopes than in the gullies.

Prior to the main analyses, the OTU composition data of the intact and disturbed communities obtained from the paired sample were combined within each collection year, and the influence of the sample collection year on the composition was assessed by PERMANOVA. No significant difference in the composition was observed between the years (Pseudo-*F*=2.309, *P*=0.061), indicating that annual variations between the two sets was minimum. Accordingly, the data collected in the two years were combined in subsequent analyses.

**Fig. 2:**
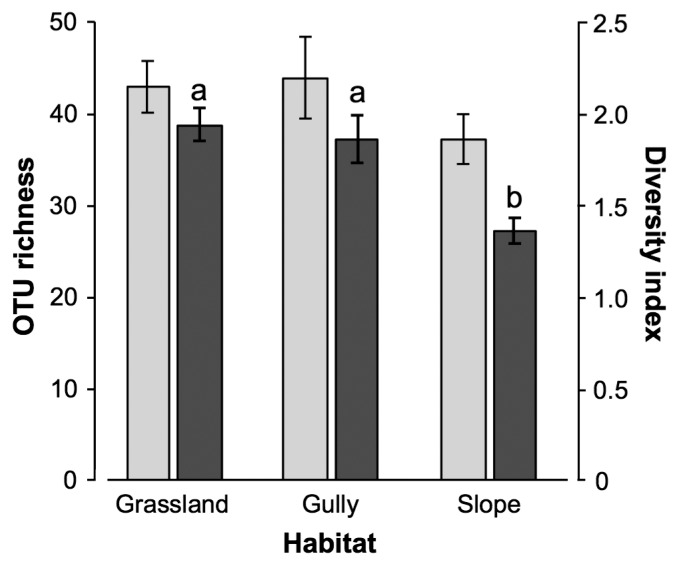
OTU richness (light grey) and Shannon-Wiener diversity indices (dark grey) of arbuscular mycorrhizal fungal communities in the rhizosphere of *Miscanthus sinensis* grown in grassland, gullies, and slopes in Mt. Tarumae. The community composition data of the intact and disturbance treatments from paired samples were combined before calculations. Values represent the mean±SE (*n*=10). Different letters indicate significant differences (Tukey’s HSD test, *P*<0.05).

**Fig. 3:**
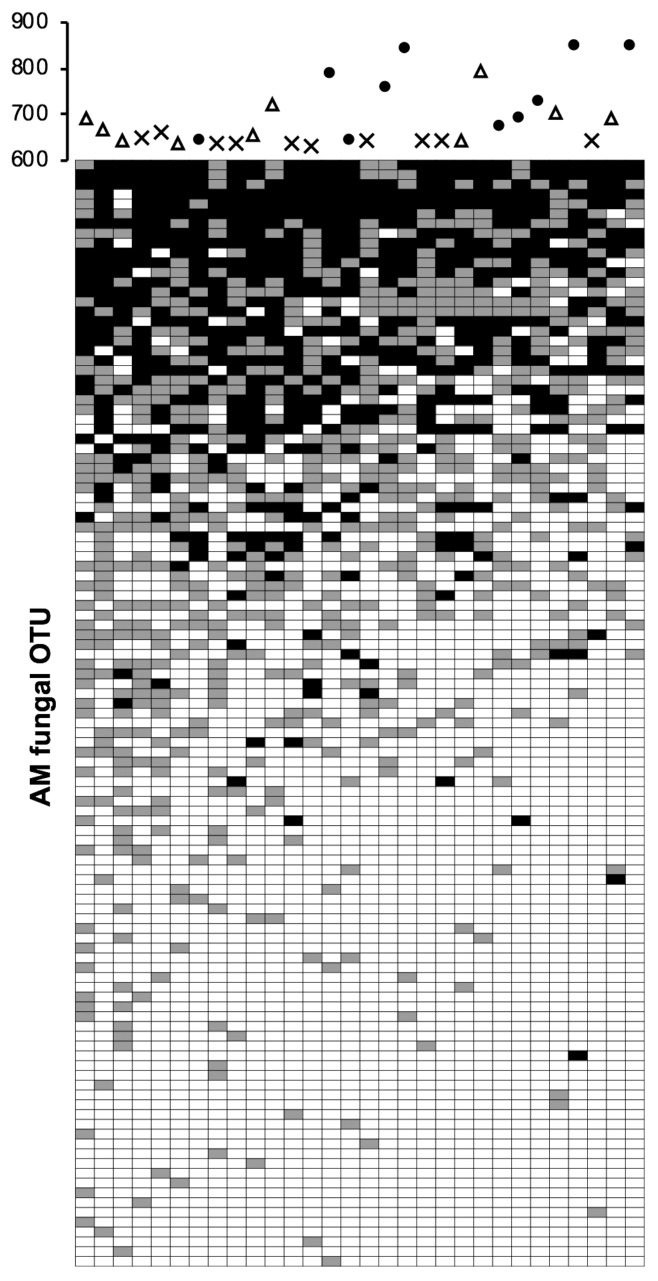
Nestedness structure of arbuscular mycorrhizal fungal communities in the rhizosphere of *Micanthus sinensis* grown in Mt. Tarumae. The community composition data of the intact and disturbance treatments from paired samples were combined and converted to presence-absence data, and columns (soil samples) and rows (AM fungal OTUs) were sorted by richness (number of OTUs) and occurrence (number of soil samples), respectively. Grey and black cells indicate the OTUs that were detected either in the intact or disturbance treatment and those that were detected in both treatments, respectively. Significance of nestedness: NODF_column_=73.69, z=11.70 (*P*<0.001); NODF_total_=58.61, z=8.72 (*P*<0.001). The scatter plot on the top of the matrix indicates elevation (Y axis) and habitat type: crosses, grassland; open triangles, gully; closed circles, slope.

Correlation analysis between AM fungal diversity and environmental factors indicated that OTU richness decreased with increasing elevations and increased with larger colony diameters of *M. sinensis* (Table 1). Diversity indices also decreased with increasing elevations and increased with higher soil pH. OTU richness did not significantly differ among the three habitats, whereas diversity indices were lower in the slope habitat than in the grassland and gully habitats (Fig. 2). Mantel test revealed that elevation (*r*=0.243, *P*=0.007) and soil phosphate (*r*=0.116, *P*=0.042) were significant environmental factors that drive the communities (Table S6). PERMANOVA indicated that community compositions were significantly differentiated among the habitats (Pseudo-*F*=2.437, *P*=0.01). Since these factors drive not only community compositions but also diversity, changes in compositions were concurrent with those in diversity, which indicated that there is either nestedness or anti-nestedness (*e.g*., species turnover) structure among the communities. To test this idea we constructed a matrix (Table S7) and performed nestedness analysis; significant nested structure was observed among the columns (NODF_column_=73.69, Z=11.70, *P*<0.001) as well as in the whole matrix (NODF_total_=58.61, z=8.72, *P*<0.001) (Fig. 3). This result implies that species (OTU)-poor communities (*i.e*. those at higher elevations and in the slopes) were subsets of species (OTU)-rich communities (*i.e*. those at lower elevations and in the grassland and gullies). The matrix further suggested that the OTUs that occurred in the intact and disturbed treatments, that is, disturbance-tolerant (or unresponsive) OTUs, were widely distributed across the ecosystem, which was subsequently tested.

### Impact of soil disturbance

**Fig. 4:**
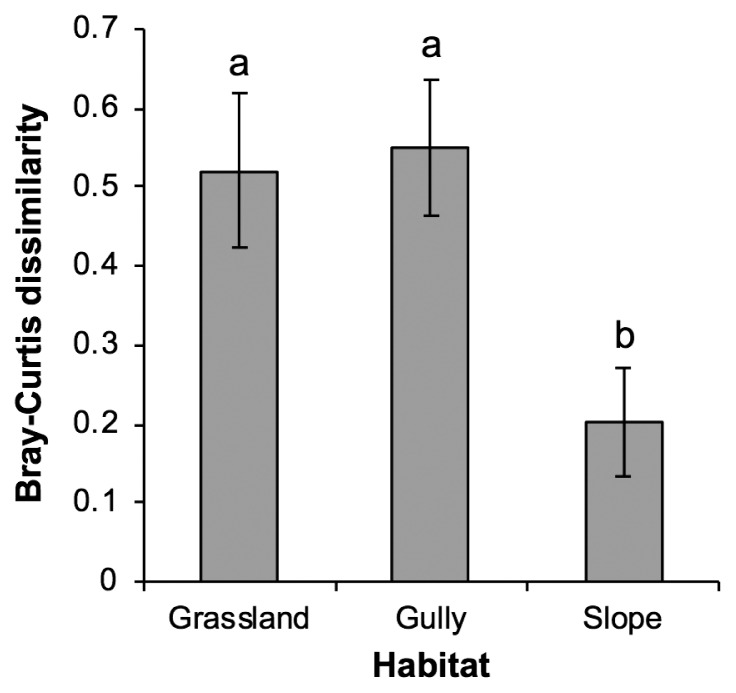
Bray-Curtis dissimilarity indices between the intact and disturbed communities of arbuscular mycorrhizal fungi in the rhizosphere of *Miscanthus sinensis* grown in grassland, gullies, and slopes in Mt. Tarumae. The index is employed as a measure of the impact of soil disturbance. Values represent the mean±SE (*n*=10). Different letters indicate a significant difference (Tukey’s HSD test, *P*<0.05).

Bray-Curtis dissimilarity indices between the intact and disturbed communities from paired samples were initially calculated, and correlation analysis between the indices and the environmental/habitat factors was performed to assess the impact of the disturbance on their compositions; elevation was the only factor that showed a negative correlation with the indices, that is, the higher the elevation, the smaller the impact (Table 2). The index was lower in the slopes than in the grassland and gullies (Fig. 4).

**Fig. 5:**
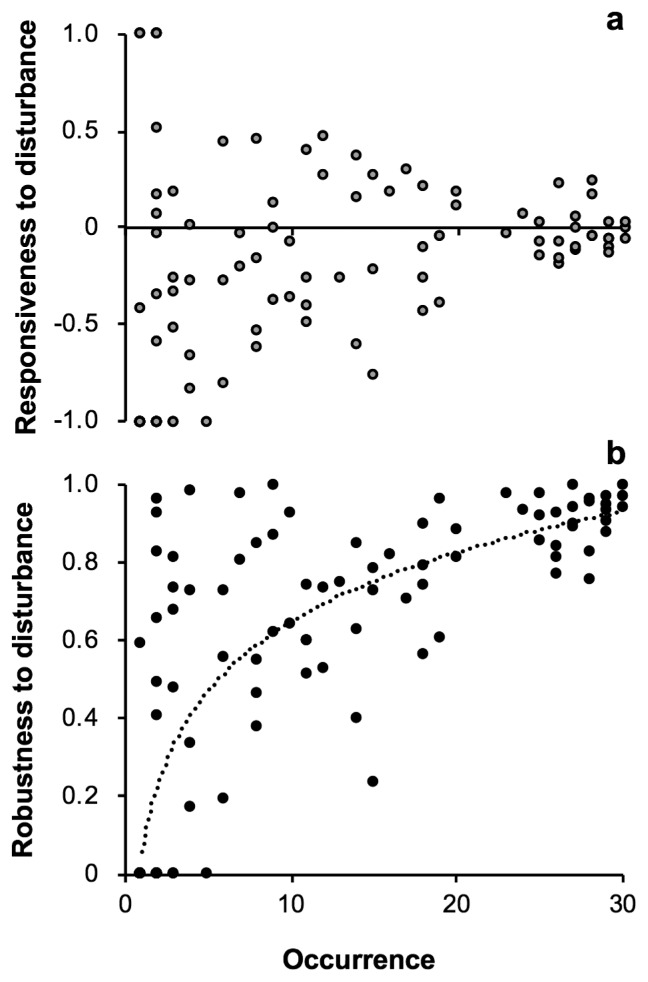
Characterization of individual arbuscular mycorrhizal fungi in the rhizosphere of *Miscanthus sinensis* in Mt. Tarumae with respect to responsiveness (a) and robustness (b) to soil disturbance. Responsiveness (RE) and robustness (RO) to disturbance represent the ratios of gained/lost sequence reads by the disturbance treatment to the total read number; RE indicates positive and negative scores for the OTUs that gained and lost sequence reads, respectively, whereas RO indicates [1–|RE|]. Occurrence is the number of paired samples in which the OTU occurred. RO showed a negative logarithmic correlation with occurrence: *r*^2^=0.671, *P*<0.001.

Individual OTUs were characterized in terms of responsiveness and robustness to soil disturbance with respect to their occurrence. The OTUs that occurred in fewer samples were more responsive to disturbance; they showed higher or lower responsiveness than those that occurred more frequently (Fig. 5a). As expected, the OTUs that occurred in more samples showed higher robustness, in which a logarithmic correlation between robustness and occurrence was observed (*r*^2^=0.671, *P*<0.001) (Fig. 5b), supporting the idea that AM fungal OTUs that are less responsive to (*i.e*. more robust against) soil disturbance are more widely distributed in the ecosystem.

## Discussion

By applying artificial disturbance to trap cultures, the present study demonstrated that soil disturbance is a major selection pressure for AM fungi associated with pioneer vegetation on the volcanic slope. The impact of disturbance on community compositions was smaller in communities on the slope at higher elevations, supporting the hypothesis that AM fungi that are tolerant to soil disturbance are selected near the crater. Furthermore, we found that communities on the slope at higher elevations (*i.e*. species-poor communities) were subsets of those in the grassland and gullies at lower elevations (*i.e*. species-rich communities); there is nested structure along a disturbance gradient in which disturbance-tolerant fungi are widely distributed in the ecosystem, that is, they are generalists.

Nested structure in AM fungal community has also been observed along soil-acidity gradients, in which acid-tolerant fungi are the generalists that are widely distributed across ecosystems (25). However, fungi in extreme environments generally evolve towards specialists that tolerate or prefer the environments, but are unable to compete in moderate environments (19). Therefore, species turnover under strong selection pressure may occur; in other words, the disappearance of sensitive fungi and appearance of tolerant fungi. The nature of generalist/specialist fungi may be interpreted according to the basic principles of biological conservation; species with a broad-niche width exhibit a high capacity to maintain fitness across harsh environments, while species with a narrow-niche width are at a higher risk of extinction under stressed conditions (6, 13). Based on the significance of AM fungal association in the establishment of vegetation in harsh environments (16, 32, 36), we propose that community nestedness in AM fungal community in volcanic ecosystems has significant implications for the resilience of vegetation after disturbance, *e.g*., by eruption and erosion; a broad distribution of disturbance-tolerant fungi enables rapid mycorrhization of plants, which secures rapid regeneration of vegetation.

Elevation represents both physical and climatic factors and is an ecologically important driver for animal, plant, and microbial communities (27), including soil fungal communities (37) and AM fungal communities (18, 30, 40). However, the difference in elevations between the forest limit (630 m a.s.l.) and vegetation limit (850 m a.s.l.) in Mt. Tarumae is only 220 m. This suggests that the influence of climatic factors on the development of vegetation gradient, as well as on the differentiation of habitats, may be minimum, and physical factors, such as soil disturbance and water availability, may drive vegetation distribution and habitat differentiation in the ecosystem. Inclination angles may be an important factor for soil stability in volcanic ecosystems, *e.g*., greater angles have higher potential for surface run-off, which is the main driver for soil disturbance (*i.e*. erosion) (12). In Mt. Tarumae, this physical (*i.e*. microgeographic) factor may be one of the primary drivers for the differentiation of habitats, and, thus, fungal communities.

The impact of soil disturbance on AM fungal communities has been demonstrated in various ecosystems, *e.g*., coastal sand dunes (24), grassland (17, 29), arable land (6, 45), and shrubland/forests (17). Microorganisms are generally sensitive to disturbance and not readily recoverable (1, 44). Among AM fungi, those that require a longer period to grow are less competitive or become extinct after soil disturbance (39) and were designated as disturbance-sensitive fungi in the present study. On the other hand, a global study revealed that anthropogenic disturbance did not always decrease AM fungal diversity; for example, at sites where natural diversity was low, disturbance increased diversity (17). In a coastal dune ecosystem in which the zonal distribution of vegetation between the seaward (frequently disturbed) and landward (stable) slopes has developed, soil disturbance increased AM fungal richness in landward soil, suggesting that potential richness in the soil is high due to the immigration of AM fungal propagules from the other side of the slope (24). In fact, the occurrence of some new OTUs after disturbance was observed in the present study, although they were rare OTUs (Fig. 5a). These results suggest that not only fungal sensitivity/tolerance to disturbance, but also potential richness/diversity need to be taken into consideration when assessing the impact of disturbance on AM fungal communities. In this context, one interpretation for the larger shifts in community composition after soil disturbance in the grassland and gullies is that potential richness/diversity in soil were higher due to the immigration of propagules from neighboring habitats.

The absolute and/or relative abundance of propagules may also affect the responsiveness of the fungi to disturbance. Soil disturbance did not decrease mycorrhizal colonization in a pasture soil in which propagule density of the fungi was high, but halved colonization in soils collected from forests and heathlands in which propagule densities were low (23). The negative impact of soil disturbance on mycorrhizal colonization may be reduced with increases in the propagule density of the fungi (34). These findings suggest that the resilience (*i.e*. rate of recovery) of the fungi after disturbance depends on propagule density. Therefore, the robustness of generalist fungi to soil disturbance observed in the present study may be interpreted not only by their tolerance to mechanical disturbance, but also by the abundance of propagule in the ecosystem. Furthermore, vegetation was only patchily distributed in the slope habitat; in other words, individual plants are isolated from each other (Fig. 1), which may limit the migration of propagule among patches. Accordingly, potential richness/diversity may be low in the slope habitat, and this may be one factor for the robustness of communities to disturbance in the habitat.

In the present study, the species-distribution pattern of AM fungi in a volcanic ecosystem has been demonstrated with respect to soil disturbance as selection pressure. Nested structure has been developed along the elevation gradient, in which generalist fungi that are robust against disturbance were enriched near the crater. Traits involved in disturbance tolerance are of interest. They may produce abundant spores, which enables rapid recolonization in the roots, and/or be capable of curing hyphal networks rapidly after disturbance; these are traits of the “ruderal” fungi defined in the trait-based framework for the life history of AM fungi (9). The finding that disturbance-tolerant fungi are generalists raises the new question of how they maintain competitiveness under non-stress conditions, in which the cost for tolerant traits may have a negative impact on their competitiveness. One possibility is that generalist fungi minimize the costs for tolerant traits in moderate environments (25), *e.g*., through rapid changes in the frequency of nucleotypes (5). The isolation and characterization of disturbance-tolerant/sensitive fungi are necessary to examine this hypothesis and, furthermore, to understand the role of these fungi in the establishment and resilience of vegetation in either naturally or anthropogenically disturbed environments.

## SUPPLEMENTARY MATERIAL





## Figures and Tables

**Table 1:** Relationships between arbuscular mycorrhizal fungal diversity in the rhizosphere of *Miscanthus sinensis* and environmental factors in Mt. Tarumae.

Factor	OTU richness		Shannon-Wiener index	
	
	*r*	*P*	*r*	*P*
Elevation	−0.457	**0.010**	−0.584	**<0.001**
Colony diameter	0.384	**0.035**	0.320	0.084
pH	0.091	0.629	0.370	**0.040**
Truog-P	0.160	0.398	0.127	0.501
Ca	0.045	0.812	0.194	0.303
Mg	0.115	0.543	0.118	0.532
K	0.010	0.956	0.004	0.980
CEC	0.134	0.475	0.015	0.936
NH_3_	0.179	0.341	0.113	0.549
NO_3_	0.051	0.784	0.267	0.153
Total N	0.135	0.475	0.801	0.673

**Table 2:** Relationships between Bray-Curtis dissimilarity indices between the intact and disturbed communities of arbuscular mycorrhizal fungi and environmental factors in Mt. Tarumae.

Factor	*r*	*P*
Elevation	−0.421	**0.021**
Colony diameter	0.277	0.137
pH	−0.058	0.758
Truog-P	0.079	0.677
Ca	−0.022	0.905
Mg	0.111	0.557
CEC	0.213	0.256
K	0.255	0.173
NH_3_	0.289	0.121
NO_3_	0.219	0.244
Total N	0.264	0.157
