# Supplementary material for: Nestedness in Arbuscular Mycorrhizal Fungal Communities in a Volcanic Ecosystem: Selection of Disturbance-tolerant Fungi along an Elevation Gradient
Source: Microbes Environ. 2019 Aug 14;34(3):327–33. doi: 10.1264/jsme2.ME19073 (PMC6759341; doi:10.1264/jsme2.ME19073)
Supplement: Supplementary file 2 [file 34_327_s2.pdf]

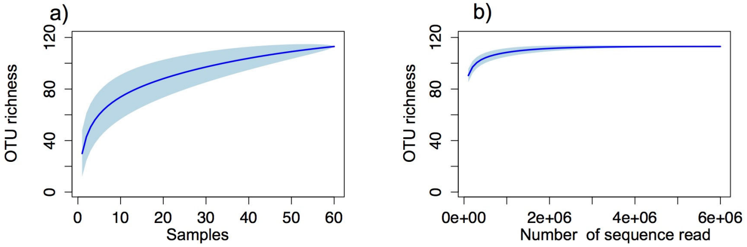

Fig. S1 Rarefaction curves constructed from number of sample (a) and number of sequence read (b). The grey area represents 95% confidence interval
